# Dyspepsia Forestalled and Resisted; or Lectures on Diet, Regimen, and. Employment

**Published:** 1832

**Authors:** 


Art. VIII.—*Dyspepsia Forestalled and Resisted; or Lectures on Diet, Regimen, and Employment. Delivered to the Students of Amherst College.* By Edward Hitchcock, Professor of Chemistry and Natural History in that Institution.

In searching for a definition to characterise the Lord of this lower world, philosophers have differed greatly in the point they have assumed as the basis of their classification.

If, in the absence of a better appellation, we were to call him a *Physic-taking animal*, we presume the species would so generally respond to the name, that the rare exceptions would serve only to give force to the general rule. The instances of men who have died without taking physic are, we venture to assert, far more rare than of those who lengthen out their span to twice the period allotted to man by the inspired penman. Scarcely has the little infant made his appearance into this world of disease, than the business of drug-taking commences; nor is the case better if he has the good fortune to make his entrance under the auspices of those whose happy experience has taught them to look upon the whole business of doctoring as a regular system of fraud and plunder. The practice thus happily commenced at the opening of life, is vigorously prosecuted through the whole term of mortal existence, until it is finally brought into requisition, with all its vain pomp and circumstance, to grace the closing scene, and carry the victim to the grave with decency. This final tribute to the Doctor's art is one that is almost never omitted, and however unceremoneously he may have been denounced during life, the mouldering remains of the poor mortal could scarcely repose in peace beneath the clods of the valley, if the rights of Esculapius were not celebrated over his dying pillow.

Is this tendency to disease, an evil inseparable from humanity, the tax that man pays for his intellectual superiority? We trow not. Nature has bestowed upon man a power of resisting the effects of noxious agents, and a facility of accommodating himself to the most injurious extremes, that is possessed by no other animal. His liability to disease, therefore, can be explained only by the fact, that he alone, of all other animals, pursues a regular system of cultivating his sensual appetites for the sordid pleasure of gratifying them. We are compelled, therefore, to make the reluctant, and somewhat mortifying acknowledgement that our honorable profession lives upon sensuality—that it thrives upon intemperance, and grows fat upon gluttony.

Since the importance of our profession is such that it cannot well be dispensed with, we are not unwilling it should be supported at the expense of those who seem to be born only to consume earth's fruits and die; but we should like to see the honorable few who stand forth as the representatives of the moral and intellectual dignity of our race, free from the ravages of that lurking and insidious Proteus, called dyspepsia, which under one form or another, is assaying the constitution of every good liver in this country, and we might say, throughout the world. Good old Seneca's maxim, "He who lives according to nature is never poor, while he who lives according to fashion, is never rich," does not require the aid of a figure to make it strictly applicable to health. Temperance and industry will enable man to bear the vicissitudes of every clime where there is any thing worth living for, and without them the greatest profusion of the gifts of Providence can never make life worth the possession.

We know of no subject upon which a manual placed in the hands of every body, would be more useful than one upon the subject of temperance—temperance we mean not in drinking—not in eating only, but in every thing—in our labor and our rest—in our study and our amusements. We have no doubt that 99 out of 100 of the actual diseases that infest the human race may be traced to intemperance under some shape. Is a man heavy, or dull, or lazy, or low spirited, or irritable, or passionate, or sick? he may depend upon it that he is habitually or occasionally intemperate, habitually or occasionally guilty of something which a rational man ought to be ashamed of,—either in eating or drinking, smoking cigars or chewing tobacco, or lying in bed in the morning, or something else which he ought to be ashamed to avow.

Among the heathen moralists temperance was a cardinal virtue—a part of their religion. It would be very desirable if its importance were equally well appreciated in our degenerate days. Believing that if it were, the cause of private happiness would be incalculably advanced, we cannot do otherwise than recommend this little work to general attention. F.

